# Real-Time Spaceborne Synthetic Aperture Radar Float-Point Imaging System Using Optimized Mapping Methodology and a Multi-Node Parallel Accelerating Technique

**DOI:** 10.3390/s18030725

**Published:** 2018-02-28

**Authors:** Bingyi Li, Hao Shi, Liang Chen, Wenyue Yu, Chen Yang, Yizhuang Xie, Mingming Bian, Qingjun Zhang, Long Pang

**Affiliations:** 1 Beijing Key Laboratory of Embedded Real-Time Information Processing Technology, Beijing Institute of Technology, Beijing 100081, China; libingyi_bit@bit.edu.cn (B.L.); yuwenyue@racobit.com (W.Y.); yangchen@bit.edu.cn (C.Y.); xyz551_bit@bit.edu.cn (Y.X.); 2 Department of Electronic Engineering, Tsinghua University, Beijing 100084, China; 3 Beijing Institute of Spacecraft System Engineering, Beijing 100094, China; bianmingming2008@163.com (M.B.); ztzhangqj@163.com (Q.Z.); 4 School of Information Engineering, Communication University of China, Beijing 100024, China; panglong@cuc.edu.cn

**Keywords:** synthetic aperture radar (SAR), real-time processing, single FPGA node imaging processing, multi-nodes parallel accelerating technique

## Abstract

With the development of satellite load technology and very large-scale integrated (VLSI) circuit technology, on-board real-time synthetic aperture radar (SAR) imaging systems have facilitated rapid response to disasters. A key goal of the on-board SAR imaging system design is to achieve high real-time processing performance under severe size, weight, and power consumption constraints. This paper presents a multi-node prototype system for real-time SAR imaging processing. We decompose the commonly used chirp scaling (CS) SAR imaging algorithm into two parts according to the computing features. The linearization and logic-memory optimum allocation methods are adopted to realize the nonlinear part in a reconfigurable structure, and the two-part bandwidth balance method is used to realize the linear part. Thus, float-point SAR imaging processing can be integrated into a single Field Programmable Gate Array (FPGA) chip instead of relying on distributed technologies. A single-processing node requires 10.6 s and consumes 17 W to focus on 25-km swath width, 5-m resolution stripmap SAR raw data with a granularity of 16,384 × 16,384. The design methodology of the multi-FPGA parallel accelerating system under the real-time principle is introduced. As a proof of concept, a prototype with four processing nodes and one master node is implemented using a Xilinx xc6vlx315t FPGA. The weight and volume of one single machine are 10 kg and 32 cm × 24 cm × 20 cm, respectively, and the power consumption is under 100 W. The real-time performance of the proposed design is demonstrated on Chinese Gaofen-3 stripmap continuous imaging.

## 1. Introduction

As an important means of space-to-earth observation, spaceborne synthetic aperture radar (SAR) has the ability to collect data continuously under all weather conditions over large areas at high resolution, making it a unique instrument [1]. SAR plays an important role in disaster emergency response, environmental monitoring, resource exploration, and geographic information access [2,3,4]. Recent publications have reviewed the applications of satellite remote sensing techniques for hazards manifested by solid earth processes, including earthquakes, volcanoes, floods, landslides, and coastal inundation [5,6,7].

In 1978, NASA’s SEASAT satellites demonstrated the ability of SAR to acquire high-resolution images. Since then, many countries have launched SAR satellites and conducted research on spaceborne SAR processing. For example, the Sentinel-1 mission, including both the S-1A (launched in 2014) and S-1B (launched in 2016) satellites, was specifically designed by the European Space Agency (ESA) to acquire data and information products for applications such as the observation of the marine environment, the surveillance of maritime transport zones, the mapping of land surfaces, and the offering of support during crisis situations [8]. The TanDEM-X/TerraSAR-X (TDX/TSX) constellation is a high-resolution interferometric SAR mission of the German Aerospace Center (DLR) intended to fulfill the requirements of a global homogeneous and high-resolution coverage of all land areas, providing vital information for a variety of applications [9]. ALOS-2 (launched in 2014) is the replacement of the Japan Aerospace Exploration Agency (JAXA) L-band SAR satellite mission to ALOS [10]. The overall objective of ALOS-2 is to provide data continuity for cartography, regional observation, disaster monitoring, and environmental monitoring. The Korea Multi-Purpose Satellite-5 (KOMPSAT-5) was launched in 2013 by the Korea Aerospace Research Institute (KARI), and the RADARSAT Constellation Mission (RCM) is scheduled for launch in 2018 by the Canadian Space Agency (CSA). The SAR data are expected to be used mainly for maritime surveillance/national security, disaster management, and ecosystem monitoring [11,12].

Most of the abovementioned missions impose high demands on the real-time performance of SAR data processing. On-board processing is an efficient solution which allows higher-precision SAR data to be processed, leading to better image quality and enabling an optional image compression. This technique improves the downlink bandwidth utilization and provides rapid feedback to the radar controller. With these processed data products, decision makers can quickly plan and respond. As early as 2000, the MIT Lincoln Laboratory began a study of the implementation of real-time signal processors for SAR front-end signal processing [13]. The processors were designed, based on their own VLSI bit-level systolic array technology, to have high computational throughput and low power implementations. S. Langemeyer et al. of the University of Hannover, Germany, proposed a multi-DSP system for real-time SAR processing using the highly parallel digital signal processor (HiPAR-DSP) technique in 2003 [14]. The small volume and low power consumption of their processor make it suitable for on-board usage in compact air- or spaceborne systems. The Jet Propulsion Laboratory (JPL) has also worked to develop on-board processing. An experimental SAR processing system based on VLSI/SOC hardware was proposed [15]. A fault-tolerant FPGA (Xilinx Virtex-II Pro)-based architecture was proposed and tested using the SIR-C data [16,17]. The University of Florida developed a high-performance space computing framework based on a hardware/software interface in 2006 [18]. An FPGA serves as the co-processor/accelerator of the CPU. A near-real-time SAR processor (NRTP) was developed by the Indian Space Research Organization (IRSO) based on the Analog Devices TigerSHARC TS101S/TS201S DSP multiprocessor. On-board or on-ground quick-look real-time SAR signal processing was found to be achievable for ISRO’s RISAT-1 [19]. With the rapid development of the storage capacity and computing capacity of the commercial-off-the-shelf (COTS) FPGA, the state-of-art Xilinx Virtex-6 FPGA was adopted for the entire real-time SAR imaging system in 2013 [20]. In recent years, the graphics processing unit (GPU), with its large computing power, has also been used for real-time SAR processing [21].

As indicated by the development of on-board SAR real-time processing, building a high-performance SAR real-time processing platform for space deployment is hampered by the hostile environmental conditions and power constraints in space. The FPGA, ASIC, DSP, CPU, and GPU are, to some extent, superior with respect to real-time processing. Although the GPU has a high processing power, its large power consumption makes it unsuitable for the harsh conditions of spaceborne on-board processing. The CPU and DSP take advantage of their design flexibility; however, they cannot provide enough FLOPS per watt, which leads to a bottleneck in their potential applications. Benefiting from its customized design, the ASIC can provide sufficient processing power and high computation ability; however, in implementing an ASIC for SAR imaging, the large-scale, complicated logic design requires a longer development period. The FPGA has made great progress in terms of on-chip storage resources, arithmetic logic resources, and hardware-software co-design. The FPGA can adapt to a large throughput rate and strict real-time signal processing requirements. Moreover, the structural characteristics of the FPGA make it suitable for homogeneous extension. 

In this paper, we propose a multi-node parallel accelerating system to realize an on-board real-time SAR processing system. The mainstream spaceborne SAR imaging algorithm, chirp scaling (CS), is implemented in this system. Previous researchers have imported CS algorithms to special FPGA processors. However, further optimizations are required for the existing methods to accelerate SAR imaging processing. For example, four times all-pixel FFTs are the most computation-hungry operations of CS implementation, and the efficiency burden mainly occurs in the data access after corner turning (matrix transposition) [22]. In a previous study [23], the window access mode was used to accelerate the matrix transposition, while in another study [24], ping pong buffers were used for Dual data rate (DDR) SDRAM to solve this problem. However, these approaches have various limitations of universality, e.g., two-dimensional rate mismatch and the method of complicated phase function generation, which can reduce the hardware resource utilization and meets the level of real-time imaging. We provide the following contributions to the existing research:

- Optimized mapping methodology for single-chip integration. The CS algorithm can be decomposed in to two parts. For the nonlinear part, nonlinear–operation linearization method, logic-memory optimum allocation, and hierarchical reconfiguration structure are proposed to reduce the complexity and time consumption. Two-dimensional bandwidth dynamic balance technology is introduced to achieve a good real-time performance between the linear part and transpose operations.
- Multi-node parallel accelerating technique for strong real-time requirements. By analyzing the spaceborne SAR real-time imaging processing conditions, this paper presents a parallel accelerating architecture consisting of a master node and multiple independent processing nodes with high processing performance, high real-time performance, and linear scalability features.

The rest of the paper is organized as follows: Section 2 reviews the CS algorithm and analyses the characteristic of each part of the CS algorithm. Section 3 presents a single-FPGA integration design for optimizing the CS algorithm implementation with optimized mapping strategy. In Section 4, the design methodology of the multi-node parallel accelerating system under the real-time principle is described. In Section 5, the corresponding hardware realization details and results are discussed. A comparison with related works is conducted to show the validity of our system. Section 6 concludes the paper.

## 2. Chirp Scaling (CS) Algorithm Review 

The CS algorithm is one of the most fundamental and popular algorithms for spaceborne SAR data processing. Compared to other algorithms, the superiority of the CS algorithm lies primarily in its use of the “chirp scaling” principle, in which phase multiplies are used instead of a time-domain interpolator to implement range-variant range cell migration correction (RCMC) shift [25]. As a kernel algorithm, the CS algorithm can process various modes with certain pre- or post-steps, such as the stripmap, scan SAR, spotlight, Tops, and Mosaic modes. This algorithm can also solve the problem of the dependence of the secondary range compression (SRC) on the azimuthal frequency because of the requirement for data processing in the two-dimensional frequency domain. 

**Figure 1:**
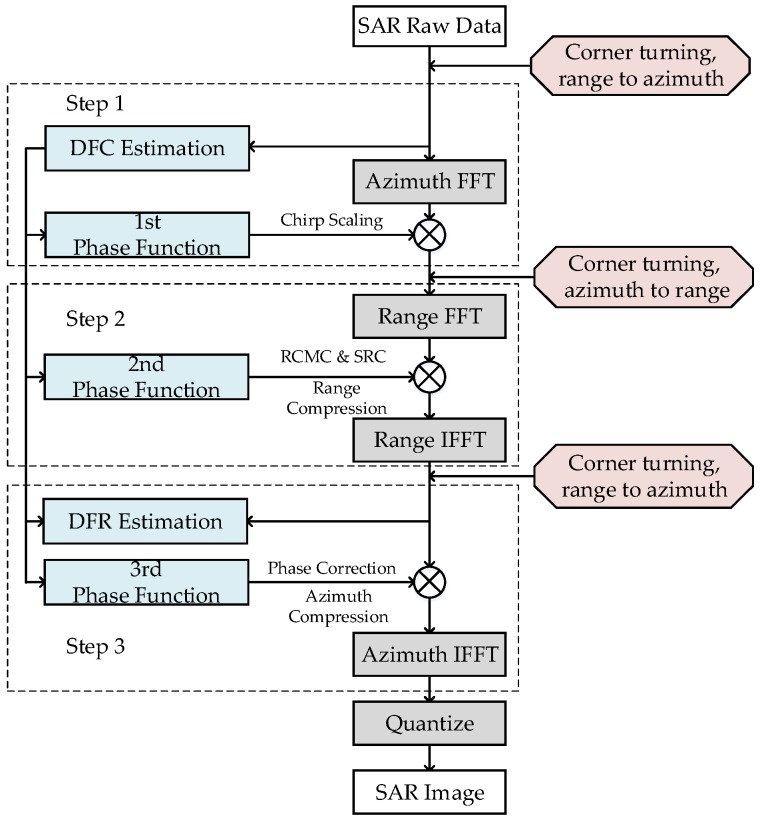
Flowchart of the chirp scaling (CS) algorithm.

The high-level block diagram of the CS algorithm based on the squint equivalent range model is illustrated in Figure 1. The algorithm mainly includes operations of four FFTs, three phase functions, and two Doppler parameters (Doppler frequency center (DFC) and Doppler frequency rate (DFR)) estimation. Note that the proposed flow introduces the raw-data-based method to ensure the precision of Doppler parameters. The steps in the CS algorithm are as follows:

First, the SAR raw data are divided into two parallel branches: one branch is transferred to the Range-Doppler domain via an FFT in the azimuthal direction, and the other branch is used to estimate DFC. DFC can be treated as a basic parameter for subsequent phase functions and DFR calculation. Second, the data are multiplied by the 1st phase function to achieve the chirp scaling, which makes all the range migration curves the same. The 1st phase function can be described as follows:

(1) $$\phi_{1}(\tau ,f_{\eta };r_{ref})=\exp[-j\pi b_{r}(f_{\eta };r_{ref})c_{s}(f_{\eta })(\tau -\frac{2}{c}r_{ref}(1+c_{s}(f_{\eta })){)}^{2}],$$

where τ is the range time, $f_{\eta }$ is the azimuthal frequency, $r_{ref}$ is the reference distance, $b_{r}(f_{\eta };r_{ref})$ is the modulating frequency in the phase center of the range direction, and $c_{s}(f_{\eta })$ is the curvature factor, expressed as follows:

(2) $$c_{s}(f_{\eta })=\frac{\sin\varphi_{ref}}{\sqrt{1-(\frac{\lambda f_{\eta }}{2v}{)}^{2}}}-1,$$

(3) $$b_{r}(f_{\eta },r_{ref})=\frac{{\left[1-(\frac{\lambda f_{\eta }}{2v}{)}^{2}\right]}^{\frac{3}{2}}\times b}{{\left[1-(\frac{\lambda f_{\eta }}{2v}{)}^{2}\right]}^{\frac{3}{2}}+br_{ref}\sin\varphi_{ref}{\left(\frac{\lambda f_{\eta }}{2v}\right)}^{2}}.$$

where λ is the wave length, *b* is the modulation frequency of the transmitted signal, $\varphi_{ref}$ and v represent equivalent squint angle and equivalent squint velocity, respectively. These variables can be described as follows:

(4) $$v=\sqrt{\frac{\lambda rf_{r}}{2}+(\frac{\lambda f_{d}}{2}{)}^{2}}$$

where $f_{d}$ represents DFC and $f_{r}$ represents DFR. Because step 1 and step 2 consider the range dimension, the initial values obtained by the ephemeris parameter can be adopted to simplify calculation. 

Third, the data are transferred to the two-dimensional frequency domain via an FFT in the range direction. Next, the data are multiplied by the 2nd phase function to complete the range compression, the SRC, and the remaining RCMC. The 2nd phase function can be described as follows:

(5) $$\phi_{2}(f_{\tau },f_{\eta };r_{ref})=\exp[-j\pi \frac{f_{\tau }^{2}}{b_{r}(f_{\eta };r_{ref})[1+c_{s}(f_{\eta })]}]\exp[+j\frac{4\pi }{c}f_{\tau }r_{ref}c_{s}(f_{\eta })],$$

where $f_{\tau }$ is the range frequency.

Next, the data are transferred to the Range-Doppler domain via an inverse FFT in the range direction. The data can be multiplied by the 3rd phase function to complete the azimuth compression and the phase correction. The DFR based on the raw data is used to refine the equivalent velocity *v* to ensure the precision of the 3rd phase function and is described as follows:

(6) $$\phi_{3}(\tau ,f_{\eta })=\exp[-j\frac{2\pi }{\lambda }c\tau (1-\sin\varphi_{ref}\sqrt{1-(\frac{\lambda f_{\eta }}{2v}{)}^{2}})+j\frac{4\pi }{c^{2}}b_{r}(f_{\eta };r_{ref})(1+c(f_{\eta }))c(f_{\eta }){\left(r-r_{ref}\right)}^{2}].$$

Finally, the inverse FFT operation in the azimuthal direction is executed to complete the CS algorithm. A visualized grayscale image can be obtained after performing the 8-bit quantization operation. 

The processing element selected for this system design is the FPGA. DDR SDRAMs are introduced into the system to act as the external storage medium for the SAR raw data. The corner turning represented in the red boxes in Figure 1 is required before each step to fit the step’s data operation dimension. For the convenience of hardware processing, the high-robustness and low-overhead algorithms called time-domain-autocorrelation (TDA) [26] and shift-and-correlate (SAC) [27] are chosen for DFC and DFR estimation, respectively. Otherwise, the SAC method would require one additional FFT operation. Thus, different mapping and implementation strategies must be designed for different operation parts.

## 3. Single-Node Field Programmable Gate Array (FPGA)-Based Architecture

### 3.1. Hardware Design Methodology

A homogeneous design reduces the complexity of the board and might be more powerful for special algorithms and decrease the system power requirements. Depending on the chosen algorithm and chassis shape of the target system, the homogeneous system can be scalable by simply adding processing nodes. In this section, we discuss the single-node FPGA design of the CS algorithm. Preparing the strategy for development requires trade-offs and integration across these multiple domains of algorithm and hardware. An efficient mapping methodology is required to ensure that the processor is able to operate without large, power-hungry computations. The process of mapping the architecture and algorithm is to progress from a correct algorithm, described at a level of abstraction that is both implementation independent and timing independent, to a system description of time-dependent, specific allocation of processing resources and the sequencing of events within those resources [28]. Compared to the multiprocessor method, uniprocessor integration can avoid the overhead of introducing multiprocessor interconnection. The real-time performance of the signal FPGA implementation mainly depends on the details of the architecture and algorithm partitioning. As described in the previous section, the CS algorithm is divided into three parts for hardware processing. The linear part, which contains a large number of rapid but repetitive computations, is the largest computational burden of real-time implementation. In addition, because the linear part is a per-image pixel operation, the communication with mass storage resource must be taken into consideration. The transpose operations affect communication overhead, which is the other factor of real-time performance. The nonlinear part consists of slower and often more irregular computations that determine the imaging quality under limited hardware resources. 

### 3.2. Nonlinear Part Mapping Strategy

In this paper, we adopt the IEEE-754 standard single precision floating-point data format to generate the phase function. A detailed description of the proposed architecture of the nonlinear part is given in this subsection. The requirement of the real-time nonlinear part design is to complete the phase function calculation before the corresponding FFT operation of each step. The estimations of the phase functions and Doppler parameters require a series of irregular and transcendental operations that can be considered a resource-intensive operation for FPGA. Adopting software computing and a per-store strategy can help increase the real-time performance of nonlinear part; however, this step requires a massive amount of storage. Therefore, a dedicated mapping method that considers the trade-off between memory and hardware resource must be designed.

#### 3.2.1. Hierarchical Decomposition Mapping Strategy and Hardware-Based Optimization Methods

**Figure 2:**
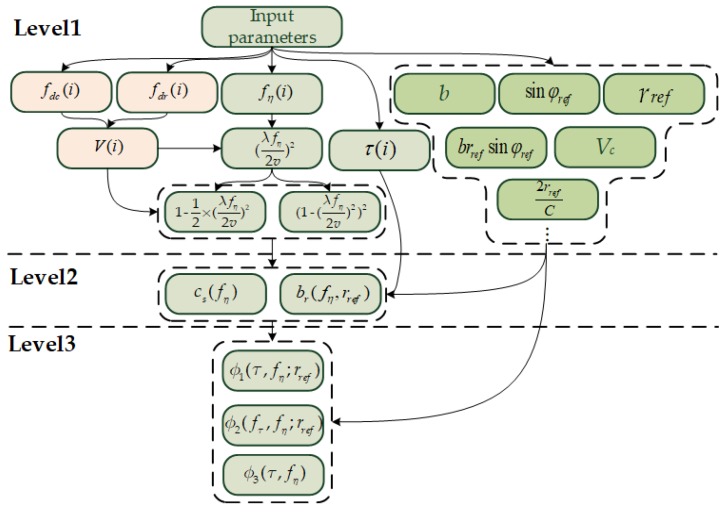
Hierarchical decomposition mapping flow.

This subsection focuses on the high-efficiency mapping strategy for phase function estimation (PFE) implementations. Because the input of the three CS factors is the basic parameter of the satellite, which does not require updating during the satellite working time, the data acquisition can be used to improve betting or storing operations. The complexity of the PFE is mainly reflected in the intermediate parameters that contain a large number of resource-hungry operations, such as prescribing and dividing. However, through the analysis, the procedure of thrice PFE is approximately the same, and it masks the possibility of parallel operations. Therefore, as shown in Figure 2, a unified three-tier mapping structure for PFE implementation, which is based on the relationship between the parameters of the calculation, is proposed.

The first level represents the procedure of fine-grained parameter acquisition. Different-colored represent different types of parameters, where the parameters in the green box are calculated only once and need not be updated during the imaging process. These parameters, such as *b*, $\sin\varphi_{ref}$, and *r_ref_*, as well as the calculations associated with them, are usually prepared in parallel at the beginning of the PFE steps and stored in the registers for subsequent operations. The gray boxes show the construction of the range axis τ(i), the Doppler frequency axis $f_{\eta }(i)$, and the corresponding parameter calculation. $f_{\eta }(i)$ is constructed by the Doppler center of reference position weighted by the ambiguity number of each azimuth line. The Taylor expansion method is supported to simplify the specific rooting operation and id described as follows:

(7) $$\sqrt{1-(\frac{\lambda f_{\eta }}{2v}{)}^{2}}=1-\frac{1}{2}\times (\frac{\lambda f_{\eta }}{2v}{)}^{2}$$

Therefore, resources are saved by having one shift operation replacing one rooting operation. The red boxes show the azimuth-related parameters, which must be updated only in step 3. As noted above, $f_{dc}$ and $f_{dr}$ can be used as the initial values in step 1 and step 2, and they should be refined in the calculation for velocity as (4), as described in step 3. Next, the procedure returns to the gray boxes displayed. The second level represents the calculation procedure of coarse-grained parameters $c_{s}(f_{\eta })$ and $b_{r}(f_{\eta },r_{ref})$ as indicated in (2) and (3). Because the inputs of these parameters are prepared in the first level and the operations are independent of each other, they can be calculated in parallel. The three-phase function calculation of the third level can be regarded as a simple assembly procedure when the parameters of the second level are ready. Note that the procedure of the 2nd PFE can be started at the second level because the fine-grained parameters are the same as those of the 1st PFE. 

According to the analysis of the mapping strategy, certain simple linear approximation methods and the elementary implementation scheme can be conducted. Advanced optimizations for further reducing the resource usage are as follows:

- Reasonable storage planning is introduced before implementation of the architecture design. The parameters maintained during one imaging process can be stored in registers, and the $f_{\eta }(i)$- and τ(i)-related parameters should be stored in DPRAM and accessed in a time multiplexing mode.
- The trigonometric functions, such as sin, cos, and arccos, are approximated by the CORDIC method, which is achieved by the addition iteration. Next, whole operations can be unified in addition (subtraction), multiplication and division. Assuming the computation of one addition as the benchmark T, one multiplication is approximately 4 T and one division is approximately 10 T. Therefore, the proposed design reduces the utilization of logical resources by using dividers, rooting, and CORDIC operational cores in a time multiplexing mode. Corresponding to Figure 2, one divider and rooting are instituted for each level, and one CORDIC is used for the first level.

#### 3.2.2. Reconfigurable Implementation Structure

**Figure 3:**
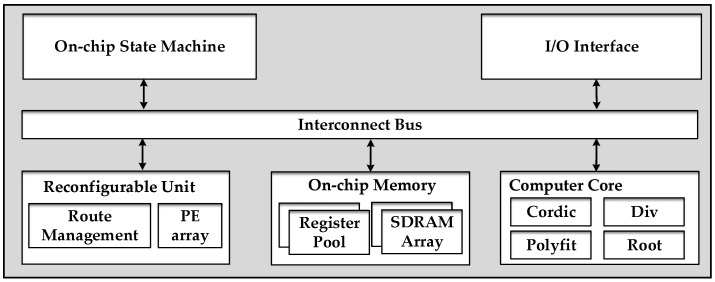
Field Programmable Gate Array (FPGA)-based architecture.

The proposed design employs the reconfigurable architecture shown in Figure 3 to achieve high utilization of the computational resources. The FPGA-based structure consists of 5 modules. The state machine unit and the I/O interface unit are responsible for system control and data input (output) management, respectively. The computer core unit and the reconfigurable unit share responsibility for computation tasks. The on-chip memory unit is divided into two groups, namely, the register pool and the SDRAM array, to store the intermediate parameters corresponding to different parameters. All modules are connected via the 32-bit user-defined and high-speed bus interface. 

**Figure 4:**
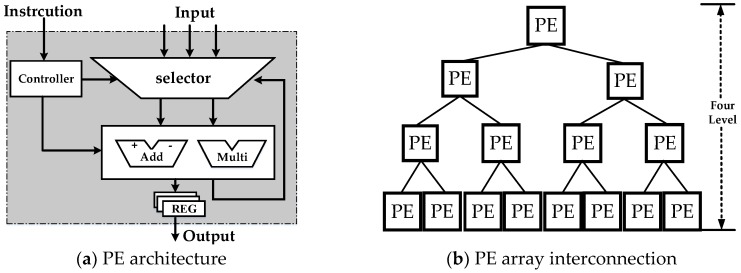
Programmable element (PE) and PE array architecture.

An isomorphism coarse-grained programmable element (PE), which is based on an instruction-driven and float-point arithmetic unit (AU), is adopted as shown in Figure 4a. The PE has a selector for four input data options: three for external inputs and one for internal computing feedback. An internal controller analyzes the instruction from route management. The PE contains three modes, namely, addition, multiplication and bypass modes, which are chosen by the instruction. Each link has the particular register, and the three modes cannot operate at the same time. Four level tree-like interconnections (Figure 4b) corresponding to the procedure of phase estimation are utilized for PE array (PEA) design. Each parent node connects all of the children nodes. Every PE can become a secondary root node and output the result independently to fit the specific scheduling task. 

#### 3.2.3. Accuracy and Time-Consumption Analysis

**Figure 5:**
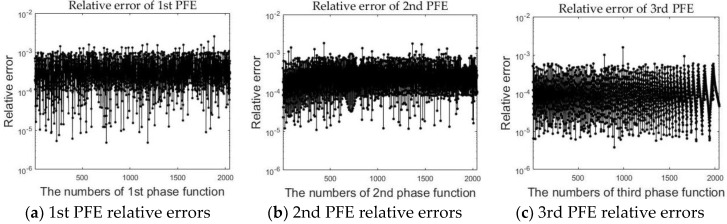
The relative errors between hardware and software computation.

The accuracy of nonlinear part estimation should be taken into consideration. The bit-level accurate simulation method based on an advanced programming language (Matlab) provides a benchmark to gauge the performance of the nonlinear part. Because the software allows a simulation result of each step of the algorithm to be provided, it is suited for verifying the validity of each PFE result perfectly. In this paper, the 16,384 × 16,384 point target image was chosen for the experiment. We chose the Xilinx XC6VSX315T FPGA as the prototype platform and adopted ISE 14.3 as the development tool. Figure 5 shows the relative errors between hardware and software computation. As described in reference [29], we extract 1 value out of every 8. Each point of Figure 5 represents the maximum relative errors of range or azimuth lines. Each value represents the maximum error for each factor line. Using the above optimized methods can result in a certain loss of accuracy, but the relative error, which is between 10^−3^ and 10^−5^, can be tolerated in high-resolution imaging. 

**Figure 6:**
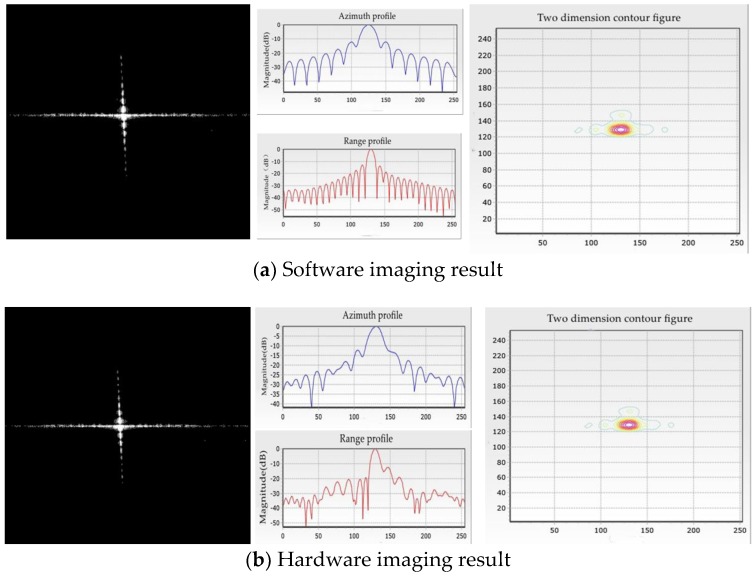
Point target imaging result with no window.

Figure 6 shows the no-window imaging result of the proposed approach and MATLAB—visually, the imaging result shows little difference between the two figures. The proposed design is also supported for window functions in range and azimuth compression stages. Additional memory can be reserved to build lookup tables for window functions. This paper chooses 5 order Taylor window with −30 dB PLSR, as well as standard Hamming window, for comparison. Table 1 shows the point target imaging quality assessment results with different situations. The peak sidelobe ratio (PSLR), integrated sidelobe ratio (ISLR), and spatial resolution (RES) are commonly adopted to evaluate the imaging quality [30]. The degradation of PSLR and ISLR between proposed and MATLAB is less than 2.5 dB, which mainly occurred by factor-extraction and linear-approximation. The result shows that the proposed design appears to be able to provide sufficient accuracy for point target imaging.

For further analysis of the influences of real-time performance, the time consumption of three PFE procedures is given in Table 2. For each PFE procedure, the time delay is less than 10 µs. Because the 16,384 × 16,384-point FFT operation delay is at the millisecond level, the PFE procedures can be considered as real-time processing and do not affect the parallelism of the linear part.

### 3.3. Linear Part Mapping Strategy

The linear part mainly includes FFTs, multiplications, and corner turning. Three corner turning (matrix transposition) operations are equivalent to the range or azimuth data access modes corresponding to each step. The proposed design introduced the sub-matrix cross-mapping method proposed in [22] to improve the efficiency of corner turning. In this manner, the two-dimensional data access can be of high bandwidth with a limited on-chip memory resource. Through introducing the timing analysis of the critical path from the perspective of the hardware structure design, the single-node optimized pipeline is obtained to instruct the actual hardware system implementation. 

#### 3.3.1. Data Access Pattern

**Figure 7:**
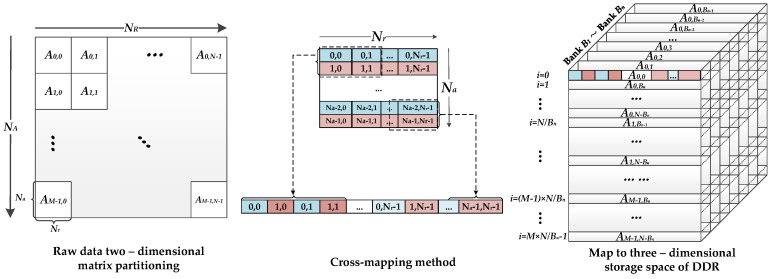
Cross-mapping method.

The principle of the cross-mapping method is to optimize and balance the range and azimuth data access rate. Each SAR raw datum is sent the DDR three-dimensional memory space according to the specific mapping rules. The main procedure of this technology (shown in Figure 7) is given by the following two steps:

1. Divide the *N_A_* × *N_R_* raw-data matrix into *M* × *N* sub-matrices of *N_a_* × *N_r_* order. To improve the mapping performance of the sub-matrix, the number of sub-matrix factors should be equal to the contained elements of each DDR row. The addressing mapping rules of raw-data matrix *A*(*x*, *y*) to sub-matrix *S*(*a*, *b*) can be described as follows:
  (8) $$\left\{ \begin{array}{ll} x\;=\;mN_{a}+\;a & 0\leq a\leq N_{a}\\ y\;=\;nN_{r}+\;b & 0\leq b\leq N_{r} \end{array}\right.$$
  where *x* and *y* represent the range and azimuth original position of raw-data matrix, respectively, and *m* (ranged in 0 to *M* − 1) and *n* (ranged in 0 to *N* − 1) determine which sub-matrix the factor is mapped to.
2. Map each sub-matrix into the three-dimensional storage array of the DDR according to the cross-mapping method. The data processing is on a bank-by-bank to rank-by-rank basis. Four factors of two lines of sub-matrices as a group are cross-mapped to the DDR row space.

Each factor in a sub-matrix corresponds to a position in the DDR memory space. Assume that the factors are mapped to the positions *D*(*i*, *j*, *k*) (coordinate of row, column, and bank) of DDR, the address mapping rule can be described as follows:

(9) $$\left\{ \begin{array}{ll} i\;=\;\left\lfloor \left(mN+n)/B_{n}\right)\right\rfloor & 0\leq i\leq MN/B_{n}-1\\ j\;=\;\left\lfloor \left(a/2\right)\right\rfloor \times 2+2b+a\;mod\;2 & 0\leq j\leq N_{a}N_{r}-1\\ k\;=\;\left(m+n\right)\;mod\;B_{n} & 0\leq k\leq B_{n}-1 \end{array}\right.$$

Simultaneous (5) and (6), the mapping rules of *A*(*x*, *y*) to *D*(*i*, *j*, *k*) can be described as follows:

(10) $$\left\{ \begin{array}{l} i=\left\lfloor (mN+n)/B_{n})\;\;\right\rfloor \\ j\;=\;\left\lfloor \left(\left(x-mN_{a}\right)/2\right)\right\rfloor \times 2+2(y-nN_{r})+\left(x-mN_{a}\right)\;mod\;2\\ k=\left(m+n\right)\;\;mod\;B_{n} \end{array}\right.$$

The data can be accessed from any location of DDR memory space, according to (7). However, considering that read and write accesses to the DDR are burst oriented, *l* × *N* samples (*l* means the burst length) should be continuously stored to or loaded from the DDR during *N* clock cycles. For example, according to the cross-mapping method, if the *l* value is 4, then two lines of data (range or azimuth) can be accessed simultaneously; if the *l* value is 8, then two lines of range data or four lines of azimuth data can be accessed simultaneously. Since each step of SAR imaging is line-based processing, it must configure a sufficient quantity of parallel caches and FFTs to fit the data operation. 

#### 3.3.2. Two-Dimensional Bandwidth Timing Analysis

Theoretically, each range line or azimuth line FFT operation is independent of the others in the SAR imaging system. In other words, the higher the degree of parallelism of the FFT (the number of FFTs can be executed simultaneously), the better the real-time performance. However, the number of processors is determined based on the computational throughput requirements, which are restricted by the bandwidth of the FFT operations, PFE operations, and DDR-FPGA communication. The one-dimensional relationship can be described as follows:

(11) $${\left[tB_{DDR},kB_{inner\_memory}\right]}_{\min}={\left[nB_{FFT},B_{PFE\_\min}\right]}_{\min}.$$

where $B_{DDR}$ is the DDR actual effective bandwidth and $B_{inner\_memory}$ is the equivalent bandwidth of the FPGA memory, which is used to cache the data between the DDR and FPGA. $B_{PFE\_\min}$ is the minimum bandwidth of the three phase function estimation operations and can be considered a real-time operation, as described above. *k* represents the amount of FPGA internal cache memory required for a specific DDR access mode. Ideally, as described above, *k* is determined by the burst length *l* of DDR and different storage methods. $B_{FFT}$ represents the bandwidth of the FFT operations, and *n* is the parallelism of FFTs. The simulation results show that the processing delay of the PFE operations is approximately dozens to hundreds of cycles. $B_{DDR}$, $B_{inner\_memory}$ and $B_{FFT}$ can be described as follows:

(12) $$B_{DDR}=\eta \rho B_{DDR\_peak}=2\eta \rho F_{DDR\_IO}W,$$

(13) $$B_{inner\_memory}=F_{inner\_memory}W,$$

(14) $$B_{FFT}=F_{FFT}W.$$

where $F_{DDR\_IO}$, $F_{inner\_memory}$, and $F_{FFT}$ represent the clock frequency of the DDR I/O, the FPGA internal memory, and the FFT co-processor, respectively. *W* is the word length of the data processed per cycle. In the all float-point system, *W* can be unified in 32. $B_{DDR\_peak}$ is the peak bandwidth of the DDR, which can be represented by the product of double $F_{DDR\_IO}$ and *W*. η represents the actual DDR bandwidth loss ratio, which is determined based on the data access mode (range or azimuth) of the DDR. ρ is the utilization of burst length *l*. Consequently, the effectiveness of the parallel processing can be described as follows:

(15) $${\left[2\eta \rho F_{DDR\_IO}W,kF_{inner\_memory}W\right]}_{\min}=nF_{FFT}W.$$

According to (15), the parallelism of co-processors is determined based on specific system features such as $F_{ddr\_IO}$, $F_{m}$, $F_{fft}$, and *W*. The two-dimensional bandwidth balance is based on one-dimensional bandwidth analysis combined with the actual system design through a reasonable design method to determine the parameters η, ρ, and k, resulting in the two-dimensional maximum *n* value.

## 4. Real-Time System Establishment

**Figure 8:**
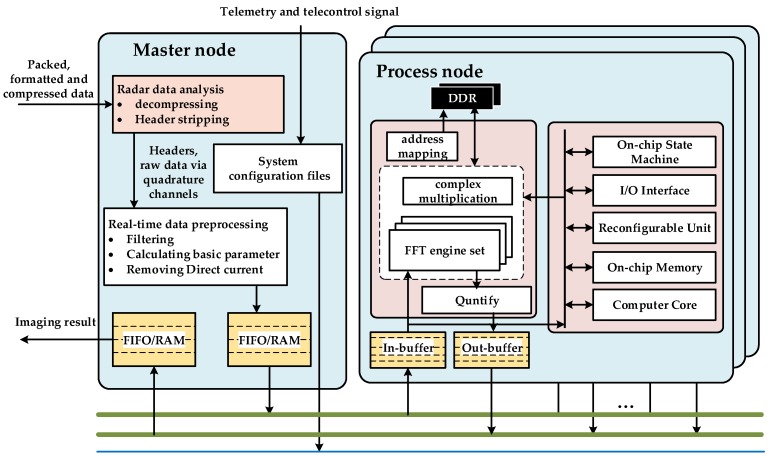
Block diagram of the multi-node parallel system architecture.

**Figure 9:**
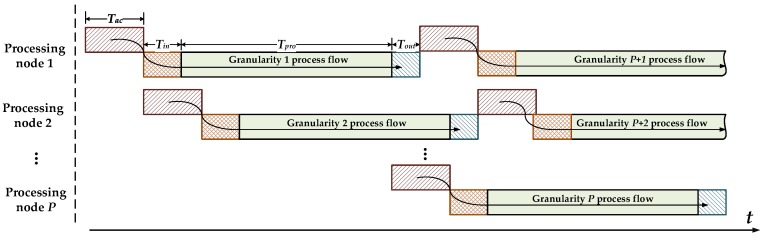
The time-sequence of multi-node processing parallel processing.

In practical application, because of the two-dimensional finite correlation of SAR data, the output of samples from radar accumulated during working state can be divided into several mutually independent data blocks, a process known as granularity. Adjacent granularity must have a certain overlap of minimum length in two directions, which are equal to samples of chirp pulse width in the range dimension and samples of synthetic aperture time in the azimuth dimension. The multi-node parallel method can be adopted for real-time processing. This method is based on the assumption that basic parameters such as attitude angles and velocity are well behaved and can be described by simple models before each granularity is processed and assembled into the SAR image granularity by granularity to produce a smooth strip image without any discontinuities. The system is mainly composed of two types of nodes: one master node and several processing nodes. The architecture is shown in Figure 8. The entire system is mounted in a high-reliability bus, which must be supported by most applications of the complex space environment since the bus is utilized only for the passing of telemetry and tele-control signal between the master node and the processing nodes; raw-data routing is handled by a high-speed parallel point-to-point custom bus for big-data runs. Conceptually, packed, formatted, and compressed data with attached header first pass to and are stored in a master node. The synchronized header data include time, attitude, beam position, and location information derived from real-time differential GPS and inertial navigation unit on-board the satellite. The header data are required for computing initial Doppler centroid, positioning, and other basic parameter calculations in real-time. After decompressing and header stripping, raw data via quadrature channels are processed by filtering and removing direct current. The master node sends a copy of the pre-processed data to processing node. Note that data input and output of processing node are handled by two completely separate buses to ensure the real-time performance of the system. The imaging result outputs for advanced product generation via the master node. The performance optimization of the proposed design is conducted at three levels:

- High performance: The proposed design works on addressing three critical requirements of high-performance spaceborne processing for SAR imaging: high computational throughput, large amount of memory, and high-speed data interconnect throughout the communication chain. Multiple processing nodes, in which instances featuring the same pipeline and available hardware resource, with corresponding independent mass storage DDR, are introduced to satisfy the first two requirements. Since the imaging procedure of each processing node is relatively independent, data reading or writing operation cannot occur at same time. Two unidirectional buses (read only and write only, processing node view) are designed not only for the high-speed data interconnects requirement, but also to avoid the bus conflict.
- Linear scalability: The standalone master node integrates the main I/O interfaces. All of the data distribution and switching control operations are conducted by the master node. The master node is responsible for optimizing I/O requests inside or outside of the system to disallow the pipeline processing nodes that lack new input data. In addition, each processing node has its own ID. In other words, the master node can distribute raw data to the processing nodes by polling the ID list. The telemetry and tele-control signal is responsible for spawning and shutting down each processing node. Modular and ID-based design allows the system architecture to have the scalability of hardware architecture; that is, the system can add (or reduce) the functionality or processing power by adding (or deleting) some of the modules.
- Real-time performance: the definition of real-time is the requirement of system that the operations, such as data transmission and computation, prescribed to be performed must be completed within a certain interval time, i.e., accumulation time of one granularity. The accumulation time can be described as follows:
  (16) $$T_{ac}=N_{A}/PRF$$
  where *PRF* is the pulse repetition frequency of the transmitter and *N_A_* represents the azimuth samples of one granularity. The data accumulation procedure occurs in the master node, and since the pre-processing is real-time, the accumulation time *T_ac_* also represents the operation delay of the master node. Therefore, the parallelism (i.e., the real-time required number of processing nodes) can be given as follows:
  (17) $$P=\left\lceil \frac{T_{ac}+T_{in}+T_{pro}+T_{out}}{T_{ac}}\right\rceil$$
  where *T_in_* and *T_out_* represent the time of raw-data input and the imaging result output of the processing node, respectively; *T_pro_* is the processing delay. To obtain clearer insight into the parallel processing strategy, the time-sequence diagram of multi-node parallel processing is shown in Figure 9: vertically, the data are assigned to the corresponding processing node in groups of *P* granularities; horizontally, each processing node handles data in turn with an interval of *P* granularities. Theoretically, the system guarantees the outputs continuously in the case of continuous inputs in real-time.

## 5. Realization of the Multi-Node Prototype Platform

To fully test and verify the functionality and performance of the proposed architecture, in this section, we first analyze and value the parameters both of single-processing node and multi-node systems, and then the prototype system and continuous imaging performances are given. 

### 5.1. Single-Node Parallel Processing Analysis 

To validate the effectiveness of the system design methodology, in this section, we establish a prototype to realize 16,384 × 16,384 stripmap SAR image processing. The standard raw data from Chinese Gaofen-3 are represented in a single precision floating-point complex form, which requires 64 bits (32 bits for the real part and 32 bits for the imaginary part) for each sample data point. DDR3 SDRAM is also introduced for the overall data storage. Table 3 shows the basic parameters of the system according to Section 3. 

**Figure 10:**
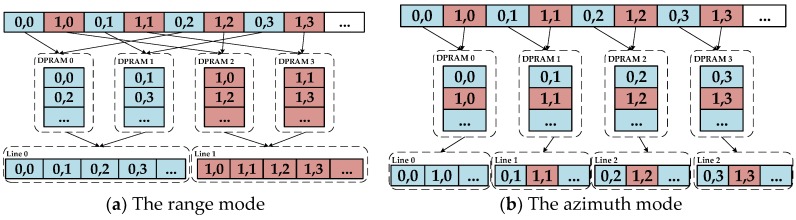
Two-dimensional data read mode.

We adopt the ping/pong memory group as caches between the DDR and FPGA; each group contains four SDRAMs with 0.125 MB of storage space. Each SDRAM is suitable for one 16 K × 64-bit data line. The burst length *l* of the DDR3 SDRAM chosen is 8. As noted above, for the range operation, the 8 × 64-bit data belong to the two range lines and four azimuth lines. Thus, to balance the two-dimensional data access rate, the DPRAM-caching method is different for range or azimuth lines, as Figure 10 shows.

In range mode, DPRAM0 and DPRAM1 cache the data of line 0 and DPRAM2 and DPRAM3 cache the data of line 1 in cross order. Thus, when the next operation must read data from DPRAM, through combining the data in the corresponding two DPRAMs, a complete range line can be obtained in the pipeline. In azimuth mode, four DPRAMs cache the data of four lines and complete azimuth data can be read out from corresponding DPRAM. In this manner, four DPRAMs can be operated in full parallel and the bandwidth of two-dimensional data access between DDR and caches can be balanced. Taking the internal data-interaction between caches and FFTs, *k* is valued as 2 in range mode and 4 in azimuth mode. Since the burst length is in full utilization, ρ is valued as 1. In this design, according to the actual measurement, η is 0.74 in two dimensions. According to (15), the number of FFTs is chosen to be 4 and 6 for range and azimuth operation theoretically. Table 4 shows a summary of the parameters related to parallel processing. The result of [22] has also been shown in Table 4 with the condition of using same basic parameters and similar hardware structure. By comparing the parameters of the corresponding positions, the proposed design has the following advantages: (1) in the range direction operation, since the cache bandwidth determines the number of FFTs, higher cache parallelism indicates that more FFT cores can be used to improve the range processing efficiency; and (2) in the azimuthal direction operation, since the DDR bandwidth determines the numbers of FFTs, full usage of the burst length also indicates that more FFT cores can be used in azimuth processing. 

### 5.2. Single-Node Imaging Result Analysis

**Figure 11:**
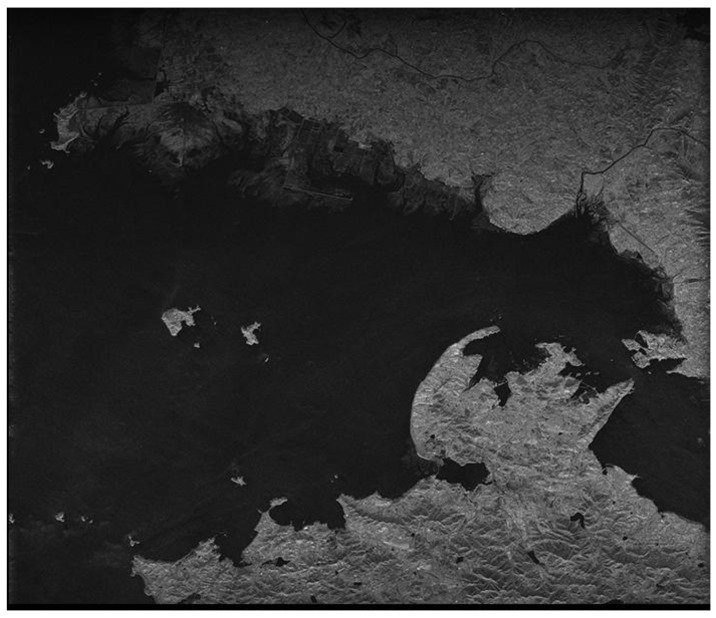
Actual scene imaging result of the system hardware.

In this paper, we chose the Xilinx XC6VSX315T FPGA as the platform of the imaging node. Because of the limits of the hardware resource, especially the storage resource, two FFTs are adopted for parallel processing. The FPGA resource occupation is shown in Table 5. Figure 11 shows the final actual scene imaging result of the proposed single-node hardware system, and the corresponding imaging quality assessment is shown in Table 6.

Through recording the numbers of clock cycles, it takes 10.6 s and 17 W for the system hardware to process SAR raw data with 16,384 × 16,384 data granularity. Table 7 shows a comparison with previous works. The time and power consumption are less than that of the related design described in [22] because the proposed system can achieve round-robin assignment in full parallel for both range direction operations. Compared with references [2,24,31,19,32], taking into account the data granularity processed, the proposed system shows advantages in both processing time and power consumption. Although [21] takes only 2.8 s to process SAR raw data with 32,768 × 32,768 data granularity, the large power consumption of the GPU is unacceptable with respect to the harsh spaceborne on-board real-time processing requirements.

### 5.3. Multi-Node System Architecture

In the proposed design, all the data transforms occur via LVDS. To approach real spaceborne processing, the procedure is designed as follows:

1. The data simulator sends the raw data of the original image to the master node via quadrature channels. The transmitted raw data are pixel-by-pixel 8: 4 compressed, and the frequency of each channel is 100 MHz.
2. Through uncompressing, filter, and direct current removing operations, the 8-bit fixed data are sent to corresponding processing nodes. Each of the quadrature channels is 11 bit × 100 MHz, including 8 bits for raw data transform, 1 bit for clock, and 2 bits for processing the node chosen ID. As noted above, since these procedures can be considered to occur in real-time, the data input time *T_ac_* is approximately 2.7 s.
3. After image processing, the result is sent back to the master node and then transmitted to the PC. The time delay of 8-bit imaging result *T_out_* is approximately 2.7 s.

The pulse repetition frequency (PRF) of Gaofen-3 satellite’s 50-km-width, 5-m-resolution stripmap mode is 1926. According to (16), it takes approximately 7.9 s to acquire one granularity of the SAR raw data. The real-time parameters of the multi-node system are shown in Table 8.

**Figure 12:**
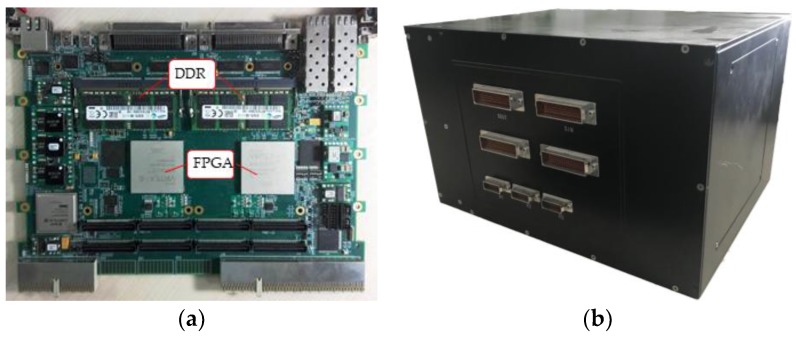
(**a**) Photographs of the board and (**b**) the prototype single machine.

Thus, we selected four processing nodes to achieve the real-time requirement. The processing board is shown in Figure 12a, which contains two independent processing nodes and the corresponding DDR SDRAMs. The other FPGA, chosen as XC5VLX30T in the prototype, is used for debugging, data flow control, and host command resolution. This FPGA communicates with two imaging FPGAs through the 2 × GTX channel. The prototype single machine is mainly composed of one master board and two processing boards, as shown in Figure 12b. The main system indicators are also shown in Table 9.

**Figure 13:**
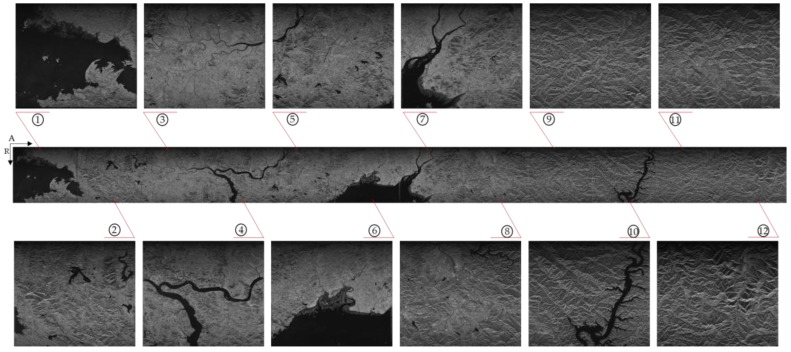
The continuous imaging results of 12 images.

Figure 13 shows the final 12 granularity imaging continuous results of the proposed prototype hardware system. The time delay of whole imaging procedure is less than 150 s. The image quality is reliable, and after a simple post-splicing processing, a complete wide image can be obtained. 

## 6. Conclusions

In this paper, to complete the on-board real-time SAR imaging processing task, a float-point imaging system using multi-node parallel acceleration technology is proposed. With an efficient mapping methodology, the whole SAR imaging procedure can be integrated in a single FPGA. To satisfy the requirement of real-time performance, we designed a prototype system with one master node and several processing nodes, and verified the performance via both a point target and an actual scene imaging quality evaluation. The efficient architecture achieves high real-time performance with low power consumption. A single-processing board requires 10.6 s and consumes 17 W to focus 25-km width, 5-m resolution stripmap SAR raw data with a granularity of 16,384 × 16,384, and the prototype single machine is suitable for continuous imaging processing.

The indicators of the single machine, such as weight, volume, and power, can satisfy the needs of spaceborne SAR imaging processing. With the development of anti-radiation reinforcement technology and system fault-tolerant technology, the proposed framework is expandable and feasible for potential spaceborne real-time SAR imaging processing.

## Figures and Tables

**Table 1:** Point target imaging quality assessment.

	Window	Azimuthal Direction			Range Direction		
		PSLR (dB)	ISLR (dB)	RES (m)	PSLR (dB)	ISLR (dB)	RES (m)
**Proposed**	**/**	−11.21	−10.00	2.93	−10.93	−9.03	2.2
**Matlab**		−12.52	−11.41	2.1	−12.61	−10.05	1.9
**Proposed**	**Taylor window**	−27.54	−21.85	4.10	−27.23	−22.34	2.45
**Matlab**		−29.74	−25.93	2.7	−28.58	−24.21	2.3
**Proposed**	**Hamming window**	−38.73	−37.21	4.61	−36.85	−34.73	3.10
**Matlab**		−40.71	−39.10	3.16	−38.32	−36.08	2.69

**Table 2:** Time consumption of three PFE procedures.

Stage	Cycles (Working Frequency of 100 MHz)
1st PFE	512
2nd PFE	512
3rd PFE	404

**Table 3:** Basic parameters of the single-node imaging implementation.

Parameter	Value
FPGA main frequency	100 MHz
*N_A_*	16,384
*N_R_*	16,384
*N_a_*	32
*N_r_*	32
*l*	8
$F_{ddr\_IO}$	400 MHz
$F_{m}$	200 MHz
$F_{fft}$	100 MHz
*W*	64 bit

**Table 4:** Summary of the parameters related to parallel processing.

Parameter	Range Direction Operation		Azimuthal Direction Operation	
	Proposed	[22]	Proposed	[22]
η	0.74	0.9375	0.74	0.74
ρ	1	1	1	0.5
*k*	2	1	4	4
$B_{ddr\_peak}$	6.4 GB/s	6.4 GB/s	6.4 GB/s	6.4 GB/s
$B_{ddr}$	4.8 GB/s	6 GB/s	4.8 GB/s	2.37 GB/s
$B_{m}$	1.6 GB/s	1.6 GB/s	1.6 GB/s	1.6 GB/s
$B_{fft}$	0.8 GB/s	0.8 GB/s	0.8 GB/s	0.8 GB/s
*n*	4	2	6	2

**Table 5:** FPGA resource occupation (Xilinx xc6vlx315t).

Parameter	Value
Number of slice registers	134,259 (34%)
Number of LUTs	122,467 (62%)
Number of block RAMs/FIFOs	499 (67%)
Number of DSP48s	387 (28%)

**Table 6:** Actual scene imaging quality assessment.

Parameter	Value
MSE	23.3
PSNR (dB)	30
SSIM	0.99
γ (dB)	4.98

**Table 7:** Comparison with previous works.

Works	Year	Schemes	Data Granularity	Working Frequency	Power Consumption	Processing Time
Proposed	2017	FPGA	16,384 × 16,384	100 MHZ	17 W	10.6 s
[22]	2017	FPGA + ASIC	16,384 × 16,384	100 MHZ	21 W	12.1 s
[2]	2016	FPGA + Microprocess	6472 × 3328	/	68 W	8 s
[21]	2016	CPU + GPU	32,768 × 32,768	/	>330 W	2.8 s
[24]	2015	Multi-DSP	4096 × 4096	100 MHZ	/	2.178 s
[31]	2012	CPU + ASIC	1024 × 1024	100 MHz	10 W	/
[19]	2008	Multi-DSP	4096 × 4096	100 MHZ	35 W	13 s
[32]	1998	ASIC	1020 × 200	10 MHz	2 W	/

**Table 8:** The real-time parameters of the multi-node system.

Parameter	Value (s)
*T_ac_*	7.9
*T_in_*	2.7
*T_pro_*	10.6
*T_out_*	2.7
*P*	4

**Table 9:** The structuring indicators of the prototype single machine system.

Indicator	Value
Weight	10 kg
Volume	32 cm × 24 cm × 20 cm
Power	<100 W
